# Systematic observation‐based diagnosis of atrioventricular nodal reentrant tachycardia with a bystander concealed nodoventricular pathway

**DOI:** 10.1002/joa3.12976

**Published:** 2023-12-15

**Authors:** Koichi Nagashima, Mitsunori Maruyama, Yoshiaki Kaneko, Satoshi Sakai, Takayuki Sekihara, Tetsuma Kawaji, Hidehiro Iwakawa, Yasuyuki Egami, Chisato Ota, Satoshi Nagase, Tetsuo Yagi, Keisuke Suzuki, Hidehira Fukaya, Hironori Nakamura, Hitoshi Mori, Akiko Ueda, Kyoko Soejima, Ryuta Watanabe, Yuji Wakamatsu, Shu Hirata, Moyuru Hirata, Yasuo Okumura

**Affiliations:** ^1^ Division of Cardiology, Department of Medicine Nihon University School of Medicine Tokyo Japan; ^2^ Department of Cardiovascular Medicine Nippon Medical School Musashikosugi Hospital Kanagawa Japan; ^3^ Department of Cardiovascular Medicine Gunma University Graduate School of Medicine Gunma Japan; ^4^ Department of Cardiology Nara Prefecture General Medical Center Nara Japan; ^5^ Department of Cardiovascular Medicine Osaka University Graduate School of Medicine Osaka Japan; ^6^ Department of Cardiology Mitsubishi Kyoto Hospital Kyoto Japan; ^7^ Department of Cardiovascular Medicine Akita University Graduate School of Medicine Akita Japan; ^8^ Division of Cardiology Osaka Rosai Hospital Osaka Japan; ^9^ Department of Cardiovascular Medicine National Cerebral and Cardiovascular Center Osaka Japan; ^10^ Department of Advanced Arrhythmia and Translational Medical Science National Cerebral and Cardiovascular Center Osaka Japan; ^11^ Department of Cardiology Sendai City Hospital Miyagi Japan; ^12^ Department of Cardiovascular Medicine Kitasato University School of Medicine Kanagawa Japan; ^13^ Department of Cardiology Saitama Medical University International Medical Center Saitama Japan; ^14^ Division of Advance Arrhythmia Management Kyorin University Hospital Tokyo Japan; ^15^ Department of Cardiovascular Medicine Kyorin University Hospital Tokyo Japan

**Keywords:** atrioventricular nodal reentrant tachycardia, nodoventricular pathway

## Abstract

**Background:**

This study aimed to establish a systematic method for diagnosing atrioventricular nodal reentrant tachycardia (AVNRT) with a bystander concealed nodoventricular pathway (cNVP).

**Methods:**

We analyzed 13 cases of AVNRT with a bystander cNVP, 11 connected to the slow pathway (cNVP‐SP) and two to the fast pathway (cNVP‐FP), along with two cases of cNVP‐related orthodromic reciprocating tachycardia (ORT).

**Results:**

The diagnostic process was summarized in three steps. Step 1 was identification of the presence of an accessory pathway by resetting the tachycardia with delay (n = 9) and termination without atrial capture (*n* = 4) immediately after delivery of a His‐refractory premature ventricular contraction (PVC). Step 2 was exclusion of ORT by atrio‐His block during the tachycardia (*n* = 4), disappearance of the reset phenomenon after the early PVC (*n* = 7), or dissociation of His from the tachycardia during ventricular overdrive pacing (n = 1). Moreover, tachycardia reset/termination without the atrial capture (*n* = 2/2) 1 cycle after the His‐refractory PVC was specifically diagnostic. Exceptionally, the disappearance of the reset phenomenon was also observed in the two cNVP‐ORTs. Step 3 was verification of the AVN as the cNVP insertion site, evidenced by an atrial reset/block preceding the His reset/block in fast–slow AVNRT with a cNVP‐SP and slow–fast AVNRT with a cNVP‐FP or His reset preceding the atrial reset in slow–fast AVNRT with a cNVP‐SP.

**Conclusion:**

AVNRT with a bystander cNVP can be diagnosed in the three steps with few exceptions. Notably, tachycardia reset/termination without atrial capture one cycle after delivery of a His‐refractory PVC is specifically diagnostic.

## Graphical abstract

The three steps to diagnosing AVNRT with a bystander concealed nodoventricular pathway (cNVP) are as follows. Step 1: identification of a concealed accessory pathway; Step 2: exclusion of orthodromic reciprocating tachycardia; and Step 3: verification of insertion of the cNVP in the AVN.

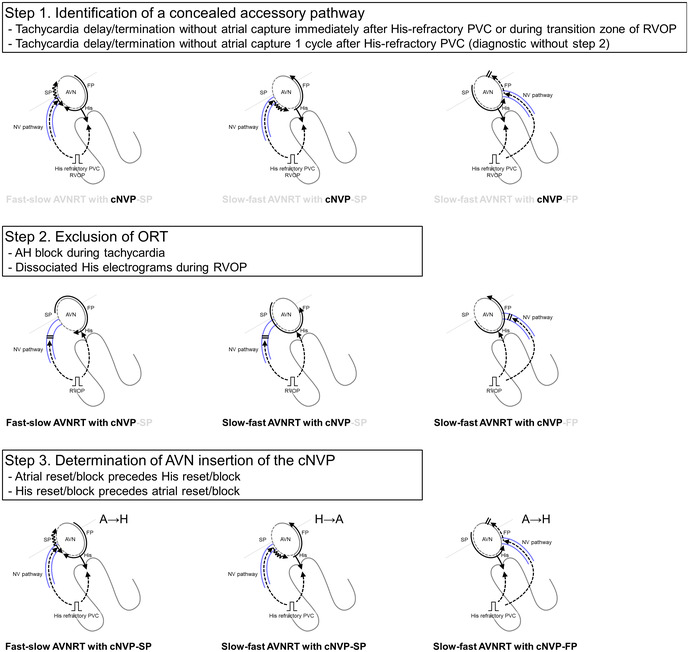

## 1 INTRODUCTION

Differentiation of orthodromic reciprocating tachycardia (ORT) incorporating a concealed nodoventricular pathway (cNVP) from atrioventricular nodal reentrant tachycardia (AVNRT) is particularly challenging and requires careful interpretation of the intracardiac electrograms.^1^, ^2^, ^3^ Furthermore, differential diagnosis is not necessarily exclusive of ORT or AVNRT; there are several reports of AVNRT with a bystander cNVP.^1^, ^2^, ^3^, ^4^, ^5^, ^6^, ^7^, ^8^, ^9^ However, criteria have not yet been developed for systematic diagnosis of AVNRT with a bystander cNVP because of the difficulty in differentiating this arrhythmia from NV‐ORT, and this lack leads to underdiagnosis. Furthermore, diagnostic observations can vary, depending on the AVNRT form, such as the slow–fast and fast–slow forms, as well as insertion of the cNVP at the AVN into the fast pathway (FP) or slow pathway (SP). We conducted a retrospective, multicenter study aimed at confirming electrophysiological observations that can, together, be used as a basis for systematic diagnosis of AVNRT with a bystander cNVP plus determination of the AVN as the site of insertion of the cNVP.

## 2 METHODS

### 2.1 Study patients

Between 2017 and 2023, we included 13 patients diagnosed with AVNRT and a bystander cNVP, and we summarized the diagnostic process; nine of them were featured in previous case reports, including two who were part of case reports written in Japanese.^2^, ^4^, ^5^, ^6^, ^7^, ^8^, ^9^ Furthermore, we included two patients who were diagnosed with ORT via a cNVP in our previous publication to assess the diagnostic performance of the sequentially scanning single premature ventricular contractions (PVCs).^2^ In these patients, PVCs were sequentially scanned with a decreasing coupling interval, ranging from the His bundle refractoriness to the effective refractory period of the right ventricle (RV, see below).

Intracardiac electrograms and measurements recorded during diagnostic pacing maneuvers were obtained from the patients' respective institutions. Data from each case were reviewed and interpreted by two independent electrophysiologists, one from the center where the patient was treated, plus one of the study authors (KN).

### 2.2 Electrophysiologic study

An electrophysiologic study had been performed with patients under conscious sedation. Then, 4‐ to 10‐pole electrode catheters were placed in the high right atrium, right ventricular (RV) apex, and His‐bundle region through the right femoral vein. Additionally, a 10‐ or 20‐pole electrode catheter was placed in the coronary sinus (CS) via the right jugular vein. Bipolar intracardiac electrograms were recorded through a bandpass filter of 30 to 500 Hz at paper speeds ranging from 100 to 200 mm/s and stored on a digital recording system (LabSystem PRO, Bard Electrophysiology; or CardioLab EP, GE Healthcare). Bipolar pacing was performed at a current strength of 10 mA and a pulse width of 2 ms. Isoproterenol was administered if the tachycardia was not inducible or atrio‐His (AH) block, ventriculoatrial VA block, or change in the VA interval were seen during the tachycardia. Diagnostic pacing maneuvers were attempted after the tachycardia, with a stable 1:1 VA relationship being confirmed.

AVNRTs were categorized according to their form as slow–fast AVNRT (His‐atrial [HA] interval ≤ 70 ms), fast–slow AVNRT (HA interval > 70 ms, AH/HA ratio < 1, and AH interval < 200 ms), or slow–slow AVNRT (HA interval > 70 ms, AH/HA ratio > 1, and AH interval > 200 ms).^10^

### 2.3 Diagnostic pacing maneuvers

To diagnose AVNRT with a bystander cNVP, two pacing maneuvers were applied. The first was delivery of sequentially scanned single PVCs, wherein the coupling interval of the His bundle refractory ventricular premature beat was gradually decreased, that is, within 30 ms of the expected His activation or recorded orthodromic His activation, to the effective refractory period of the right ventricle (RV). Presence of an accessory pathway (AP) was recognized on observation of tachycardia reset, i.e., advancement or delay of the His bundle electrogram by ≥8 ms, or of termination of the tachycardia without atrial capture.

The second maneuver was RV overdrive pacing (RVOP) at a cycle length (CL) 10 to 30 ms shorter than the tachycardia CL (TCL). RVOP was initiated when the coupling interval between the last catheter‐sensed RV electrogram and the first paced beat approximated the pacing CL. Entrainment was confirmed when the atrial CL decreased to match the pacing CL without altering the atrial activation sequence, and the tachycardia resumed when the pacing was terminated. Presence of an AP was recognized when the atrial electrogram was reset, i.e., advanced or delayed by ≥8 ms, within the transition zone of QRS morphology.^11^ An “A‐V" response after RVOP, an uncorrected/corrected post‐pacing interval (PPI) minus TCL of >125/110 ms, and absence of orthodromic His capture were confirmed to reinforce the diagnosis.^2^, ^12^, ^13^

### 2.4 Statistical analyses

Data are shown as the number of patients, number (and percentage) of patients, or mean ± SD values.

## 3 RESULTS

### 3.1 AVNRT features and location of the cNVP

**FIGURE 1:**
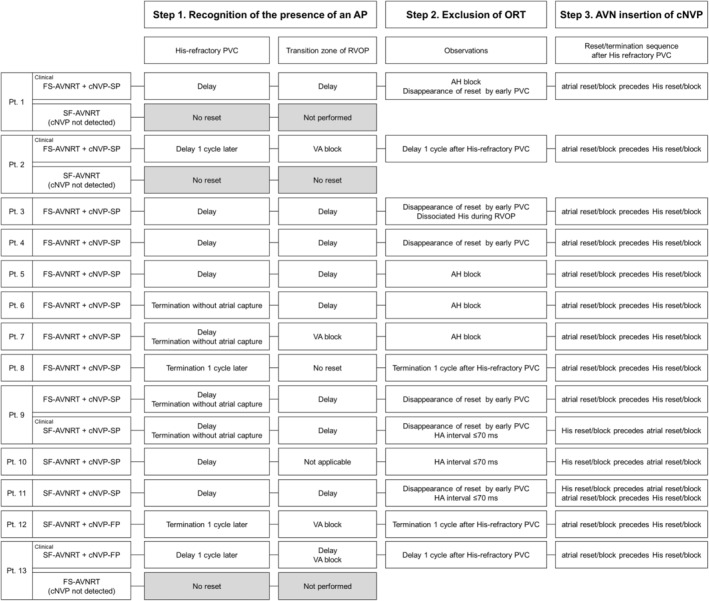
First two steps to the diagnosis of AVNRT with a bystander cNVP and the corresponding observations per study patient. A, atrium; AA, atrial‐atrial; AH, atrio‐His; AVNRT, atrioventricular nodal reentrant tachycardia; cNVP, concealed nodoventricular pathway; FS, fast‐slow; HA, His‐atrial; HH, His‐His; PVC, premature ventricular contraction; RVOP, right ventricular overdrive pacing; SF, slow‐fast; V, ventricle; VA, ventriculoatrial.

**FIGURE 2:**
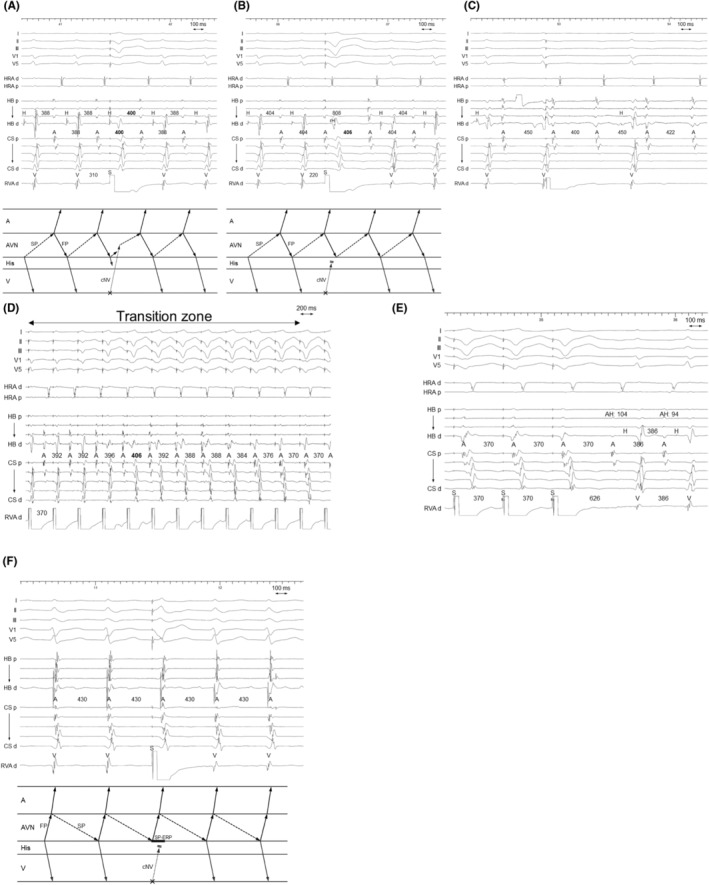
Patient 1. Intracardiac electrograms of (A–E) fast–slow AVNRT with a bystander cNVP connected to the slow pathway (SP) and (F) slow–fast AVNRT. (A) The PVC delivered during His‐refractoriness delays the tachycardia, with with a delay of the atrium preceding the His delay, indicating presence of an AP (Step 1). (B) The reset phenomenon disappears after the early PVC, which captures the His antidromically (rH). The observation indicates that both the AP and His‐bundle are outside the tachycardia circuit, which is diagnostic of fast–slow AVNRT with a bystander cNVP connected to the SP and excludes ORT (Step 2). (C) The tachycardia sustains despite occurrence of atrioHisian block, which indicates that His‐bundle is not involved in the tachycardia circuit and rules out ORT (Step 2). (D) Right ventricular overdrive pacing (D) delays the atrial electrograms during the transition zone and (E) results in a post‐pacing interval − tachycardia cycle length of 140 ms, which also supports the diagnosis. (F) The His‐refractory PVC fails to uncover the presence of an AP during the slow‐fast AVNRT, possibly due to the SP ERP. A, atrium; AP, accessory pathway; AVN, atrioventricular node; cNVP, concealed nodoventricular pathway; CS, coronary sinus; d, distal; ERP, effective refractory period; FP, fast pathway; H, His; HB, His bundle; HRA, high right atrium; ORT, orthodromic reciprocating tachycardia; p, proximal; PVC, premature ventricular contraction; RVA, right ventricular apex; S, stimulus; SP, slow pathway; V, ventricle.

**FIGURE 3:**
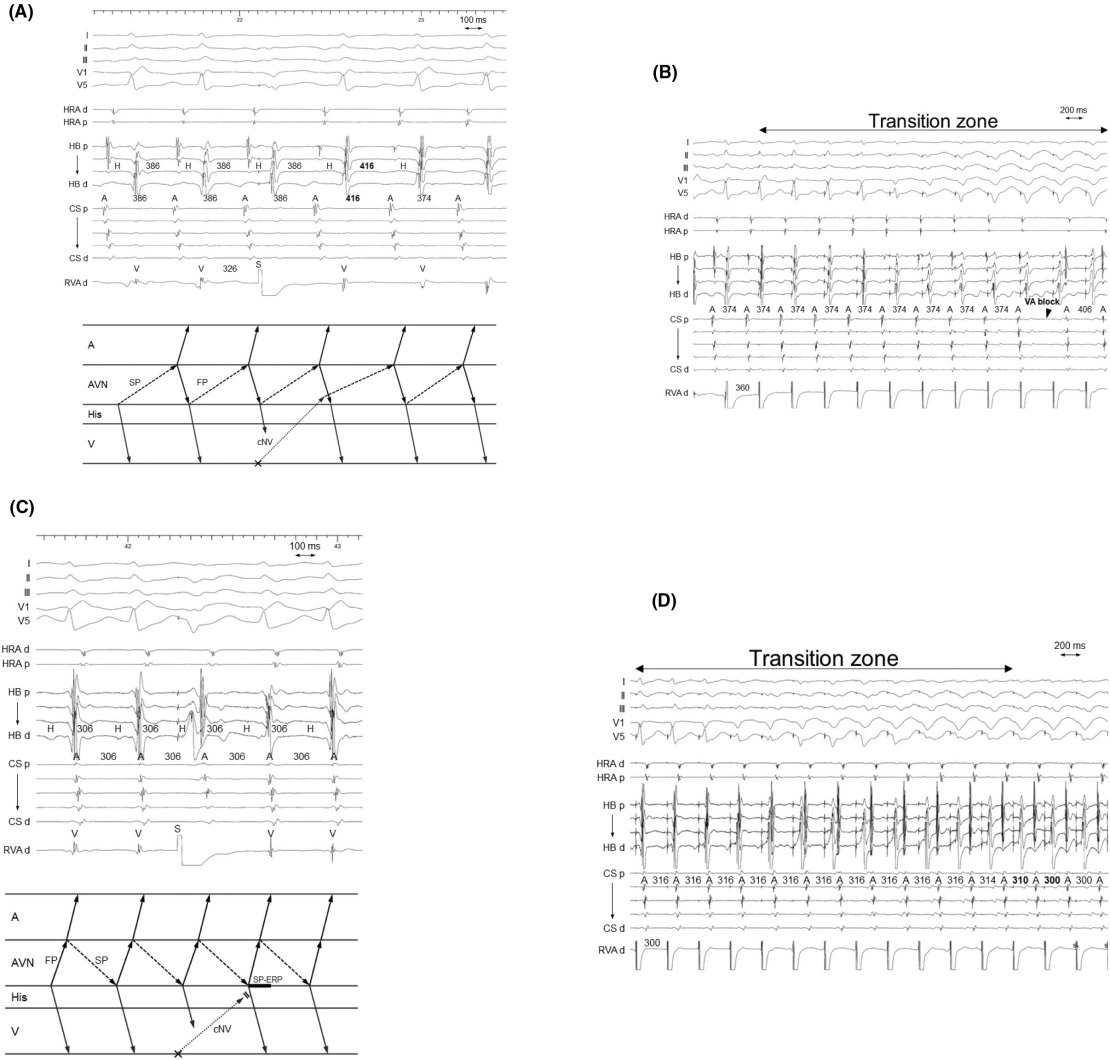
Patient 2. Intracardiac electrograms of (A,B) fast–slow AVNRT with a bystander cNVP connected to the slow pathway (SP) and (C,D) slow–fast AVNRT. (A) The premature ventricular contraction (PVC) delivered during His‐refractoriness delays the tachycardia 1 cycle later with a delay of the atrium preceding the His delay. This observation is considered diagnostic of fast–slow AVNRT with a bystander cNVP because it indicates presence of a cNVP and excludes ORT (Steps 1 and 2). (B) Right ventricular overdrive pacing results in VA block during the transition zone, which also supports the diagnosis. However, (C) the His‐refractory PVC and (D) right ventricular overdrive pacing fails to detect presence of an AP during the slow–fast AVNRT, possibly due to the SP ERP. Abbreviations are as in Figure 2.

**FIGURE 4:**
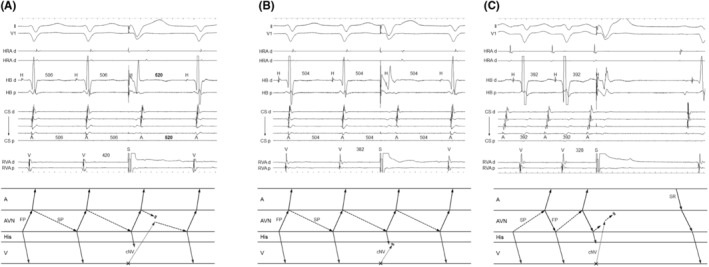
Patient 9. Intracardiac electrograms recorded during (A,B) slow–fast AVNRT with a bystander cNVP connected to the SP and during (C) fast–slow AVNRT. (A) The PVC delivered during His‐refractoriness delays the tachycardia with a delay of the His preceding a delay of the atrium, indicating presence of an AP (Step 1). (B) The reset phenomenon disappears after the early PVC, indicating that the tachycardia is sustained despite conduction block over the AP, which is diagnostic of slow‐fast AVNRT with a bystander cNVP connected to the SP and rules out ORT (Step 2). (C) In contrast, the His‐refractory PVC terminates the tachycardia without atrial capture in the fast–slow AVNRT. Abbreviations are as in Figure 2.

**FIGURE 5:**
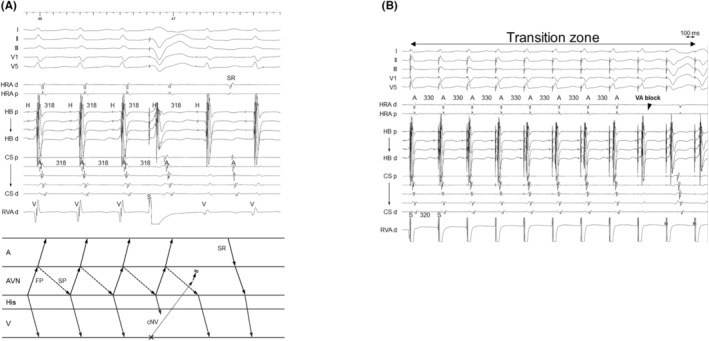
Patient 12. Intracardiac electrograms recorded during slow–fast AVNRT with a bystander cNVP connected to the FP. (A) The PVC delivered during His‐refractoriness terminates the tachycardia 1 cycle later without atrial capture. This observation is diagnostic of slow–fast AVNRT with a bystander cNVP that is connected to the FP and rules out ORT (Steps 1 and 2). (B) Right ventricular overdrive pacing also causes VA block during the transition zone, supporting presence of a cNVP. Abbreviations as in Figure 2.

**FIGURE 6:**
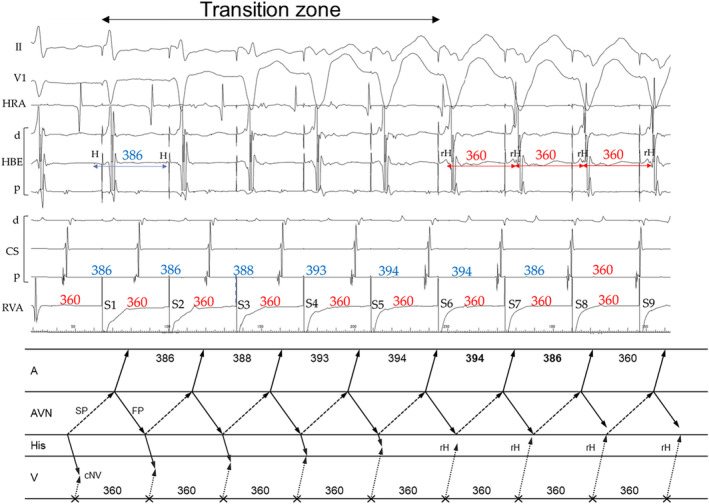
Patient 3. Intracardiac electrograms of fast–slow AVNRT with a bystander cNVP connected to the slow pathway (SP). Right ventricular overdrive pacing delays the atrial electrograms during the transition zone. Moreover, the pacing retrogradely captures the His electrogram but fails to accelerate the atrium to the pacing cycle length in two beats, indicating the dissociated His from the tachycardia. Abbreviations as in Figure 2.

Basic clinical and electrophysiologic characteristics of the 13 patients are shown in Table 1. To summarize the diagnostic process, the diagnosis of AVNRT with a bystander cNVP was determined through the stepwise observations, as detailed for each patient in Figure 1, and representative intracardiac electrograms and estimated laddergrams are shown in Figures 2, 3, 4, 5, 6. The AVNRT was of the fast–slow form in eight patients and of the slow–fast form in five patients. In addition, both fast–slow AVNRT and slow–fast AVNRT were inducible during the procedure in four patients (Patients 1, 2, 9, and 13 Figures 2, 3, 4). Thus, a total of 17 AVNRTs (10 fast–slow AVNRTs and seven slow–fast AVNRTs) were analyzed. The bystander cNVP was connected to the SP (cNVP‐SP) in 11 of the 13 patients and to the FP (cNVP‐FP) in two as described later. Three AVNRTs were found to be sustained despite occurrence of the VA block.

**TABLE 1:** Patients' clinical and electrophysiological characteristics (*n* = 13).

Age (years)	51 ± 22
Male gender	7 (46%)
*Electrophysiology findings*	
Dual AV nodal physiology	
Antegrade	8 (57%)
Retrograde	5 (38%)
Tachycardia features (*n* = 17)	
TCL (ms)	372 ± 53
Fast–slow AVNRT	10 (59%)
Slow–fast AVNRT	7 (41%)
AH block during AVNRT	4 (24%)
VA block during AVNRT	3 (18%)
His‐refractory PVC	
Delay the tachycardia	11 (65%)
Delay immediate	9 (53%)
Delay one cycle later	2 (12%)
Advance the tachycardia	0 (0%)
Termination without atrial capture	6 (35%)
Termination immediate	4 (24%)
Termination one cycle later	2 (12%)
RV overdrive pacing	
Termination	6 (35%)
PPI−TCL (ms)	173 ± 29
Corrected PPI−TCL (ms)	165 ± 26

*Note*: Values are mean ± SD or *n* (%).

Abbreviations: AH, atrio‐His; AV, atrioventricular; AVNRT, atrioventricular nodal reentrant tachycardia; PPI, post‐pacing interval; RV, right ventricular; TCL, tachycardia cycle length; VA, ventriculoatrial.

### 3.2 Diagnosis of AVNRT with a bystander cNVP

Differential diagnosis of narrow QRS tachycardia included junctional tachycardia (JT), atrial tachycardia (AT), ORT via an atrioventricular AP or nodofascicular pathway (NFP), and AVNRT with or without a bystander AP. In all cases, the diagnostic process of AVNRT with a bystander cNVP was summarized by taking the three steps described below.

### 3.3 Step 1. Identification of the presence of a concealed atrioventricular AP or cNVP

The first step was identification of the presence of a concealed atrioventricular AP or cNVP. The presence of either AP, except the NFP, was recognized on observation of tachycardia reset without any change in the atrial sequence and/or termination without atrial capture upon His‐refractory PVC that exhibited a fused QRS morphology.^2^, ^4^, ^5^, ^6^, ^7^, ^8^, ^9^ In our study, presence of either AP was recognized in 14 of the 17 AVNRTs. Tachycardia reset with delay was observed either immediately after delivery of the His‐refractory PVC (*n* = 9; Figures 2A and 4A) or 1 cycle after delivery of the His‐refractory PVC (*n* = 2; Figure 3A). Termination of the tachycardia without atrial capture was observed either immediately after delivery of the His‐refractory PVC (*n* = 4; Figure 4C) or 1 cycle after delivery of the His‐refractory PVC (*n* = 2; Figure 5A). In addition, during the transition zone of RVOP, reset of the atrial electrogram (*n* = 9; Figure 2D) or termination without atrial capture (*n* = 4; Figures 3B and 5B) was observed, which was a similar observation to recognize the presence of either AP. No advancement of the atrial electrogram was seen upon delivery of the His‐refractory PVC, but atrial advancement within the transition zone during RV OP was seen in one slow–fast AVNRT.

Such observations excluded JT, AT, and AVNRT. Moreover, AVNRT with a bystander atrioventricular AP was also excluded. However, ORT via any AP, except a cNFP, and AVNRT with a bystander cNVP remained possibilities. The ORT via a cNFP was ruled out because resetting the tachycardia with a His‐refractory PVC that exhibited a fused QRS morphology indicated ventricular involvement in the tachycardia circuit.

### 3.4 Step 2. Exclusion of ORT via an AP

The second step was exclusion of ORT. This was accomplished by demonstrating that the His bundle, AP, or both were outside the tachycardia circuit. Specifically, the AH block during the tachycardia also excluded ORT (*n* = 4; Figure 2C). Additionally, ORT was ruled out when the tachycardia reset phenomenon disappeared upon delivery of the His‐refractory PVC that was earlier than the PVC satisfying Step 1 (*n* = 1; Figure 4B), suggesting the sustainment of the tachycardia despite the AP block or of an early PVC that captured the His electrogram antidromically (*n* = 6; Figure 2B), suggesting dissociation of the His bundle from the tachycardia. However, the disappearance of the reset phenomenon upon delivery of an early PVC was also observed in the two patients with ORT via a cNVP (Supplemental Figure S1A,B). In contrast, in one patient, RVOP retrogradely captured the His electrogram but failed to accelerate the atrium to the pacing CL in two beats, which also indicated the dissociated His from the tachycardia (Figure 6). This phenomenon was not observed in any of NV‐ORT cases.

Such observations supported the diagnosis of AVNRT; (1) absence of orthodromic His or septal ventricular capture and (2) a PPI − TCL of >125 ms or a corrected PPI − TCL of >110 ms after RVOP (*n* = 9; Figure 2E).^2^, ^4^, ^5^, ^7^, ^9^, ^12^, ^13^ Specifically, (3) the HA interval ≤ 70 ms (*n* = 5; Figures 2F, 3C, 4A,B and 5A,B) supported the diagnosis of slow–fast AVNRT excluding the ORTs, as previously reported.^2^, ^4^, ^8^, ^14^, ^15^ As shown in the Supplemental Figure S1, this NV‐ORT appeared to exhibit the HA interval of ≤70 ms. However, this was attributed to an extremely long HA interval nearly equal to the TCL. Consequently, atrial electrograms overlapped with ventricular electrograms in the subsequent beat. This phenomenon was differentiated by the observation of the V‐V‐A response after RVOP, as detailed in previous publications, in contrast to the A‐V response observed in all AVNRT patients.^2^, ^16^

Notably, tachycardia reset (*n* = 2; Figure 3A) and termination without atrial capture (*n* = 2; Figure 5A) occurring 1 cycle after the His‐refractory PVC observed in Step 1 were found to rule out ORT.

### 3.5 Step 3. Verification of the AVN as the cNVP insertion site according to the reset/termination sequence after delivery of a His‐refractory PVC

The third step was to determine whether the cNVP was inserted into the AVN, i.e., into the SP or FP according to the reset/termination sequence after His‐refractory PVC. Delay of the atrial electrogram preceding that of the His electrogram or termination without atrial capture after delivery of the His‐refractory PVC was defined as the atrial reset/block preceding the His reset/block. Delay or block of the His electrogram preceding that of the atrial electrogram was defined as the His reset/block preceding the atrial reset/block.

In all nine fast–slow AVNRTs with a bystander cNVP‐SP, the atrial reset/block preceding the His reset/block was observed such as delay of the atrial electrogram preceded that of the His electrogram (*n* = 7; Figures 2A and 3A) and/or termination of the tachycardia without atrial capture (*n* = 4; Figure 4C) following the delivery of the His‐refractory PVC. In contrast, in the three slow–fast AVNRTs with a bystander cNVP‐SP, the His reset/block preceding atrial reset/block was observed such as delay or block of the His electrogram preceded that of the atrial electrogram (Figure 4A). Additionally, in one of the three slow–fast AVNRTs with a bystander cNVP‐SP, both sequences were observed; the atrial reset preceding the His reset occurred only when the earliest atrial activation shifted immediately after PVC moved from the His bundle region to the proximal coronary sinus region.^8^ A bystander cNVP‐FP was diagnosed in two cases of slow–fast AVNRT because the atrial reset/block preceded the His reset/block upon the delivery of the His‐refractory PVC, a finding that cannot be explained by a cNVP‐SP (Figure 5A).

### 3.6 Diagnostic performance in cases of multiple AVNRT forms

Both slow–fast AVNRT and fast–slow AVNRT were inducible in four patients; three with a cNVP‐SP and one with a cNVP‐FP. The cNVP‐SP was apparent during both slow–fast and fast–slow AVNRT in one of the four patients (Figure 4A–C), whereas the cNVP‐SP was apparent during the fast–slow AVNRT in the remaining three patients, the diagnostic maneuvers failed to detect the cNVP during slow–fast AVNRT in two of these three patients (Patients 1 and 2; Figures 2F and 3C,D). The diagnostic maneuvers also failed to detect the cNVP during fast–slow AVNRT in the patient with a bystander cNVP‐FP (Patient 13).

## 4 DISCUSSION

### 4.1 Main findings

**FIGURE 7:**
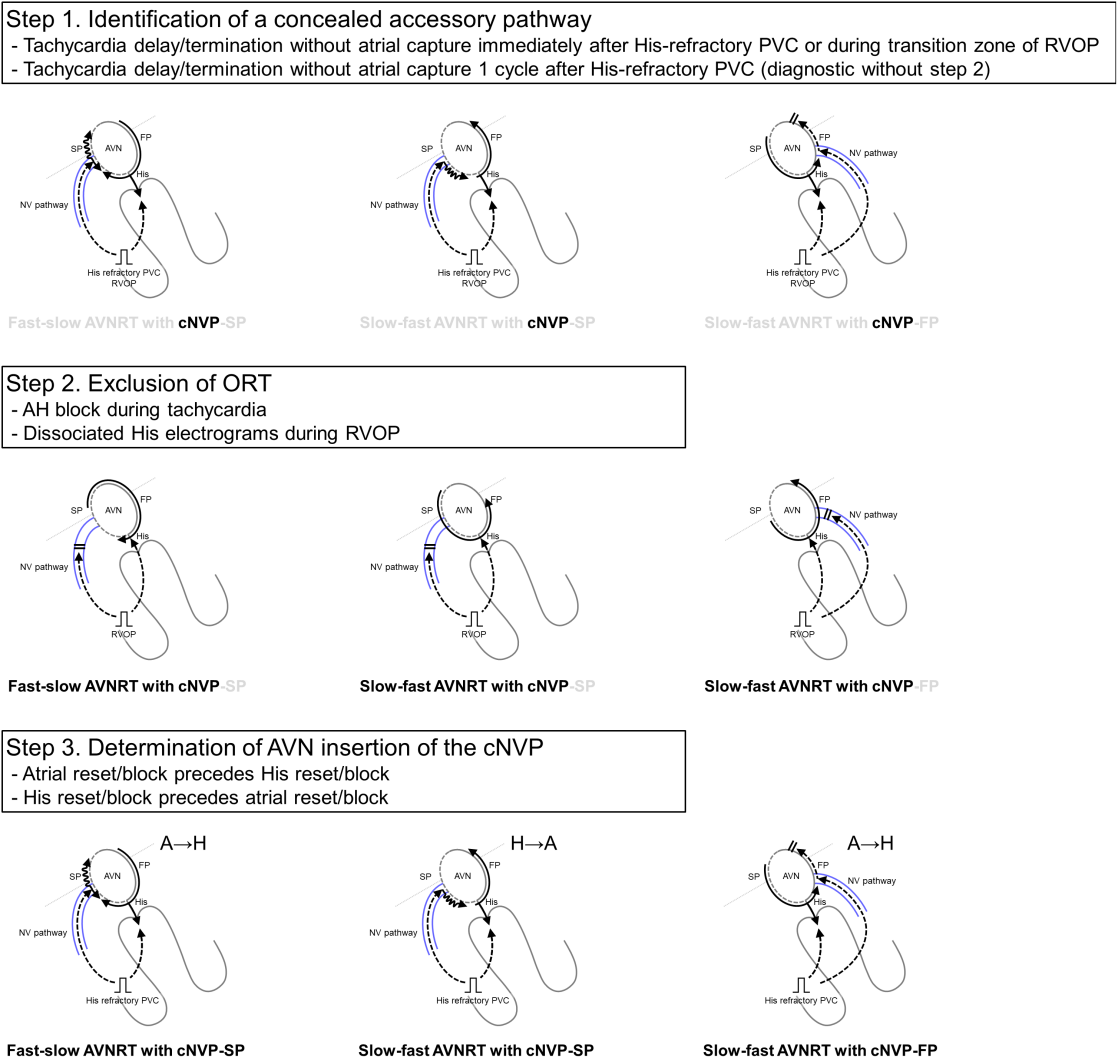
The three steps to diagnosing AVNRT with a bystander concealed nodoventricular pathway (cNVP). Step 1: identification of a concealed AP by delivery of a His‐refractory PVC or right ventricular overdrive pacing; Step 2: exclusion of orthodromic reciprocating tachycardia (ORT), proving that the His‐bundle is outside the circuit based on the observations such as atrio‐Hisian block during the tachycardia or the dissociated His electrograms during right ventricular overdrive pacing; and Step 3: verification of insertion of the cNVP in the AVN based on the AVNRT form and reset/termination sequence. Notably, a delay or termination without atrial capture 1 cycle after the His‐refractory PVC is specifically diagnostic of AVNRT with a bystander cNVP. Abbreviations as in Figure 1.

AVNRT with a bystander cNVP was exclusively diagnosed by means of the following three steps (Figure 7): (1) identification of a concealed atrioventricular AP or cNVP by His‐refractory PVC or RVOP; (2) exclusion of ORT based on the dissociation of the His‐bundle from the tachycardia, which is evidenced by AH block during the tachycardia or by RVOP; and (3) verification of the AVN as the cNVP insertion site per AVNRT form and the reset/termination sequence. Notably, reset or termination of the tachycardia without atrial capture 1 cycle after the His‐refractory PVC was specifically diagnostic of AVNRT with a bystander cNVP. Note that the disappearance of the reset phenomenon upon delivery of an early PVC was observed not only in AVNRT with a bystander cNVP but also in a few NV‐ORT cases. Therefore, this phenomenon may not be specific for AVNRT with a bystander cNVP but rather an exceptional observation. Nonetheless, this phenomenon suggests the presence of cNV.

### 4.2 Clinical flow of the diagnostic Steps 1–2

Steps 1 and 2 were observed either during tachycardia or commonly performed pacing maneuvers such as His‐refractory PVC and RVOP. If a reset (usually manifested as a delay) in tachycardia occurred without any change in the atrial sequence upon delivery of a His‐refractory PVC or during the transition zone of RVOP (Step 1), and if an AH block during the tachycardia or a dissociated His electrogram during RVOP was observed (Step 2), then the diagnosis was established.

In Step 2 of the AVNRT with a bystander cNVP, the tachycardia reset phenomenon disappeared upon delivery of the His‐refractory PVC that was earlier than the PVC satisfying Step 1 or an early PVC that captured the His electrogram antidromically. This phenomenon suggests the sustainment of the tachycardia despite the AP block or dissociation of the His bundle from the tachycardia. However, this phenomenon was also observed in ORTs via a cNVP. This occurrence can arise because the reset may be masked if the prematurity of the PVC equals the delay in ORT caused by the PVC. Therefore, proving the dissociated His bundle from the tachycardia by a single PVC is challenging. Although this phenomenon suggests the presence of AVNRT with a bystander cNVP, it may not be specific for the diagnosis. Given this limitation, there remains a small chance of diagnosing ORT in four patients, although the observations such as absence of orthodromic His and a long uncorrected/corrected PPI − TCL were consistent with a diagnosis of AVNRT with a bystander cNVP.

Furthermore, reset or termination of the tachycardia without atrial capture 1 cycle after the His‐refractory PVC is specifically diagnostic (Steps 1–2). The reset or termination of the tachycardia was attributable to a conduction block that could occur anywhere from the activation of the cNVP or the SP to the subsequent activation of the His bundle. Theoretically, it is implausible that His‐refractory PVC resets or terminates the tachycardia without affecting the next His electrogram.^9^

### 4.3 Reset/termination sequence and verification of the AVN as the cNVP insertion site (Step 3)

In all cases of fast–slow AVNRT with bystander cNVP‐SP, the atrial reset/block preceded the His reset/block upon the delivery of the His‐refractory PVC, in contrast to the reverse in slow–fast AVNRTs with the cNV/SP. These observations can be attributed to the difference in conduction direction over the SP between the two forms of AVNRT. Since the SP acts as the retrograde limb in fast–slow AVNRT, the His‐refractory PVC conducts to the SP retrogradely through the cNVP, leading to the resetting of the atrial electrogram before that of the His electrogram (Figure 7). However, in slow–fast AVNRTs, the SP acts as the anterograde limb, leading to the His‐refractory PVC conducting to the SP anterogradely and resetting the His timing preceding the atrial timing (Figure 7). Similarly, in the context of slow–fast AVNRTs with cNVP‐FP, since the FP acts as the retrograde limb in slow–fast AVNRT, the His‐refractory PVC conducts to the FP retrogradely through the cNVP, leading to the resetting of atrial timing before the His timing (Figure 7). The most convincing explanation for the exceptional slow–fast AVNRT case, in which the atrial reset occurred before the His reset concurrent with a shift in atrial activation sequence, is that the PVC captured the atrium through the cNVP and the SP connected with the cNVP. This SP is distinct from the anterograde limb of the slow–fast AVNRT.^8^ Therefore, the reset/termination sequence may be useful for verification of the AVN as the cNVP insertion site especially when the atrial activation sequence remains unchanged after the PVC.

### 4.4 Diagnosis with respect to the AVNRT forms and insertion of the cNVP in the AVN

Our study addressed the potential difficulty of diagnosis of AVNRT with a cNVP with respect to SP vs FP and insertion of the cNVP in the AVN. Among the 11 patients (12 AVNRTs) in whom a cNVP‐SP was detected, the AVNRTs were of the fast–slow form in the majority (*n* = 9, 75%) of patients. Moreover, among the three patients in whom both the fast–slow and slow–fast forms were inducible, the cNVP‐SP was detected during the slow–fast AVNRT in only one patient. Interestingly, fast–slow AVNRT is uncommon, at a reported prevalence of 5% (vs. 81% for slow–fast AVNRT and 14% for the slow–slow AVNRT),^14^ we found the bystander cNVP‐SP to be more often detected in the fast–slow AVNRTs than in the slow–fast AVNRTs. This can be explained by the refractory period of the SP to which the cNVP is commonly connected. The SP is excitable at the time of His‐bundle refractoriness during fast–slow AVNRT, and the PVC can reach the cNVP‐SP connection and reset (Figures 2A and 3A) or terminate (Figures 4C) the tachycardia according to the refractoriness in the proximal segment of the SP. However, during slow–fast AVNRT, this period is immediately after the SP depolarization (Figure 2F), which is within the refractory period of the SP. Therefore, the cNVP‐SP would be less detectable during the slow–fast AVNRT. Nevertheless, the slow–fast AVNRT can be reset only if either of the following conditions is met: (1) rapid recovery from the refractoriness in SP (Figure 4A) or (2) long enough conduction time over the cNVP to recover the SP conductivity by the His‐refractory PVC.

In contrast, the bystander cNVP‐FP was detectable in the slow–fast AVNRTs. However, the cNVP‐FP was not detectable during the fast–slow AVNRT despite being detected during slow–fast AVNRT in the same patient. Similarly, this observation can be explained by the refractory period of the FP that is connected to the cNVP. The FP is excitable at the timing of the His‐bundle refractoriness during slow–fast AVNRT, and the PVC can reach the cNVP‐FP connection and reset or terminate (Figure 5A) the tachycardia according to the refractoriness in the proximal segment of the FP. In contrast, the His‐refractory PVC fails to reset the fast–slow AVNRT due to the refractory period of the FP. According to our results, presence of a cNVP can go unrecognized depending on the AVNRT form and insertion of the cNVP in the AVN. Slow–fast AVNRT with a cNVP‐SP and fast–slow AVNRT with a cNVP‐FP, in particular, may be missed.

### 4.5 Study limitations

Our conclusions must be interpreted in light of our study limitations. The study was conducted as a small, retrospective investigation. Nonetheless, we believe that our findings are clinically meaningful because they are based on meticulous analysis of the electrophysiologic characteristics of the arrhythmias and of the electrocardiographic measurements. In addition, in each case, the choice of diagnostic pacing maneuvers was dependent on the institution's or physician's preference. However, in general, the same diagnostic maneuvers, including delivery of His‐refractory PVCs and RVOP, were performed. Verification of the AVN as the cNVP insertion site according to the reset/termination sequence was not supported by the histological evidence. Further histological study would be warranted.

## 5 CONCLUSIONS

Our study showed that AVNRT with a bystander cNVP can be diagnosed by a 3‐step observation‐based process: (1) identification of an AP by delivery of a His‐refractory PVC or RVOP; (2) exclusion of ORT based on the dissociation of the His‐bundle from the tachycardia, which is evidenced by AH block during the tachycardia or by RVOP; and (3) verification of cNVP insertion into the AVN per AVNRT form and observation of the reset/termination sequence. Furthermore, reset or termination of the tachycardia without atrial capture 1 cycle after the His‐refractory PVC is specifically diagnostic of AVNRT with a bystander cNVP.

## CONFLICT OF INTEREST STATEMENT

The authors have no conflict of interest to declare.

## ETHICS STATEMENT

Approval of the research protocol: The review board of Nihon University Itabashi Hospital and the review board of each participating center approved the data collection and analysis.

## INFORMED CONSENT

Patients had consented to the use of their data for research purposes through an opt‐out method.

## Supporting information


Supplemental Figure 1.
