# Supplementary figures and images for: Systematic observation‐based diagnosis of atrioventricular nodal reentrant tachycardia with a bystander concealed nodoventricular pathway
Source: J Arrhythm. 2023 Dec 15;40(1):131–42. doi: 10.1002/joa3.12976 (PMC10848616; doi:10.1002/joa3.12976)

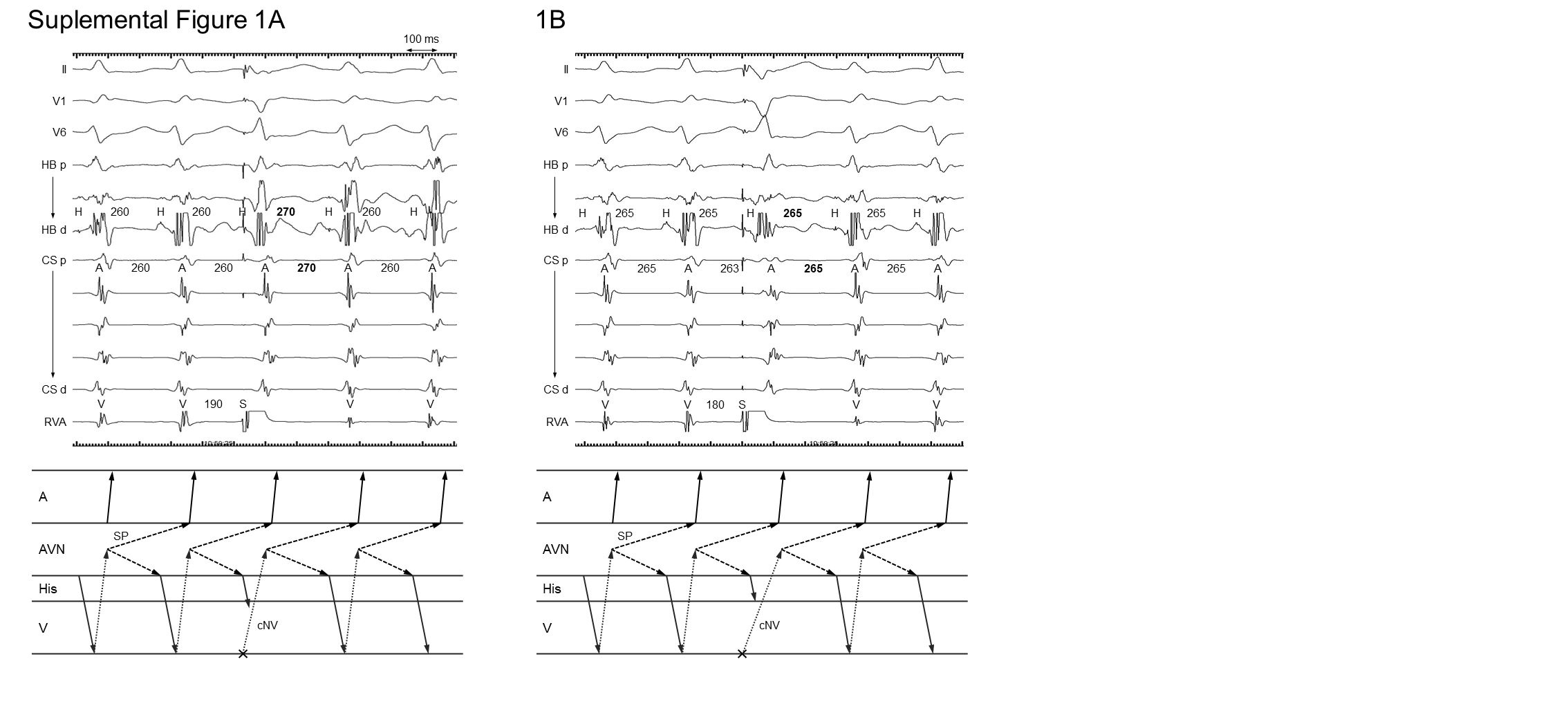

Supplement: Supplementary file 1 — Supplemental Figure 1. [file JOA3-40-131-s001.tif]
